# Impact of Stability of Enriched Oil with Phenolic Extract from Olive Mill Wastewaters

**DOI:** 10.3390/foods9070856

**Published:** 2020-06-30

**Authors:** Rosa Romeo, Alessandra De Bruno, Valeria Imeneo, Amalia Piscopo, Marco Poiana

**Affiliations:** Department of AGRARIA, University Mediterranea of Reggio Calabria, Vito, 89124 Reggio Calabria, Italy; rosa.romeo@unirc.it (R.R.); alessandra.debruno@unirc.it (A.D.B.); valeria.imeneo@unirc.it (V.I.); mpoiana@unirc.it (M.P.)

**Keywords:** macroporous resin XAD-7HP, olive mill wastewaters, oxidative stability, polyphenols, sunflower oil

## Abstract

The disposal of olive mill wastewaters is a considerable subject for the development of a sustainable olive oil industry considering their high content of pollutants. Nevertheless, the selective extraction of phenolic compounds from olive mill wastewaters represents a promising approach to obtain phenolics suitable for food enrichment. This work aimed to evaluate the efficiency of phenolic extract addition (50 mg L^−1^), used as natural antioxidant, in sunflower oil against oxidative deterioration; to this aim, XAD-7-HP resin was tested in the recovery of phenolic compounds from olive mill wastewaters. Ultra-high performance liquid chromatography was used to evaluate the single phenols contained in the extract; the most consistent amount was detected for hydroxytyrosol (834 mg 100 mL^−1^). The change in the oxidation state of fortified sunflower oil was studied by measuring physicochemical (refractive index, peroxide value and oxidative resistance to degradation) and antioxidant parameters (DPPH, ABTS and ORAC assays) during 90 days of storage. Results showed an enhancement of oxidative stability of 50% in the fortified oil compared to control.

## 1. Introduction

Olive oil production represents a very important sector for Mediterranean countries. Despite its economic importance, olive oil production is associated with some negative implications on the environment as contamination of soil, water body pollution, underground seepage and air emissions due to the large amount of waste generated [1,2]. Olive mass is composed of about 80% of olive pulp and stones, whereas the liquid and solid waste yield is greater than the oil after the production steps [3]. Specifically, the amount of produced olive mill wastewaters (OMWWs) varies from 0.3 to 1.1 m^3^ for tons of processed olives, depending on the olive oil extraction system [4,5]. OMWW disposal is a serious problem for the development of a sustainable olive oil industry owing to the high content of pollutants as heavy metals, a considerable amount of suspended solid and organic compounds. As a result of their high water solubility, the polyphenol concentration in OMWW ranged from 5 to 25 g L^−1^ [6,7]. These compounds show strong antioxidant activity, principally based on their ability to transfer the hydrogen atom of phenolic hydroxyl group to the free radicals. Phenolic compounds have potential beneficial effects with their anti-inflammatory and antimicrobial properties [8,9,10].

However, the selective recovery of phenolic substances from industrial wastes, such as OMWW, represents a valid approach for the reduction of their environmental toxicity and an opportunity to obtain high added value molecules [11,12]. Several methods for the recovery of OMWW polyphenols have been investigated, such as solvent extraction, ultrasound treatment, supercritical fluid extractions, membrane, chromatographic separations and Amberlite XAD resins, with the final aim to maximize the phenolic yield without impurities and toxic substances, and after to use these in the food industry [8,13,14,15]. Literature is available on the application of solid extraction and purification methods with the use of polymeric resin. A variety of synthetic polymer adsorbents are nowadays available for OMWW treatment, such as polystyrenedivinylbenzene copolymers and divinylbenzeneethylvinylbenzene as acrylic ester-based Amberlite XAD7 and the styrenedivinylbenzene-based XAD16 resin copolymers [16,17,18,19]. The adsorption process is characterized by a simple design and operation, low operating costs, reduction of the amount of used solvent, reduction of the operation time and separation of large amounts of bioactive compounds [14,20,21]. Moreover, the relatively inexpensive resins are durable, chemically stable and safe. In this work, Amberlite-XAD7HP was used as mean for the obtainment of a natural phenolic extract (WE) suitable for oil enrichment. XAD7HP resin exhibits a suitable pore diameter, polarity and surface area to recovery low molecular weight phenols using solvents with low toxicity and safe for human use. In addition, the potential to be reused with a low loss of absorbing capacity over time makes it useful for industrial application.

Considering the OMWW dotation of phenols with recognised antioxidant activity and health benefits, in the last few years there was an increasing interest in their recovery to re-use these as natural antioxidants. The enrichment with natural phenolic extracts of foods, beverages, edible oils, etc. (that contain a low concentration of phenolic compounds), could be a viable alternative to obtain a healthy added-value product. Sunflower oil with higher amounts of unsaturated fatty acids, in particular polyunsaturated ones, is more exposed to oxidative rancidity or autoxidation. This process reduces the nutritional and sensorial qualities and also gives undesirable chemical products, such as organic acids, aldehydes and ketones, which are harmful for human health [22]. Chemical stability depends on the chemical constituents of the oil, like antioxidants and oxidizable components [23]. Sunflower oil is usually subjected to a refining process that involves the complete loss of valuable active components with interesting nutritional-, health- and antioxidant-related characteristics [24]. Although synthetic antioxidants initially have been used with the purpose of retarding the oxidative deterioration, their implications in some diseases, such as cancer and carcinogenesis, are still controversial [25]. Thus, the tendency to use natural antioxidants has been increasing in order to increase the food quality and satisfy the demand of consumers for healthy products [26]. 

The aim of this study was to evaluate the efficiency of the phenolic extract obtained from OMWW with a solid extraction method against sunflower oil oxidative deterioration.

## 2. Materials and Methods

### 2.1. Chemicals and Reagents

Standards of phenolic compounds gallic acid (99%), vanillic acid (97%), tyrosol (97%), ferulic acid (99%), *p-*coumaric acid (98%) and *o*-cumaric acid (98%) were purchased from Fluka (Steinheim, Germany). Caffeic acid (98%), Apigenin (99%), luteolin (99%), and oleuropein (99%) were purchased from Extrasynthèse (Z. I. Lyon Nord, France). Hydroxytyrosol ((3,4-dihydroxyphenyl) ethanol) was acquired from TCI (Saitama, Japan). Verbascoside (99%) was procured from Sigma-Aldrich (St. Louis, MO, USA). The solvents used for chromatographic analysis (methanol, water, and acetonitrile) were ultra-high performance liquid chromatography (UHPLC)-MS grade (Carlo Erba, Milan, Italy). 2,2′-Azino-bis (3-ethylbenzothiazoline-6-sulfonic acid) diammonium salt (ABTS), 2,2-diphenyl-1-picrylhydrazyl (DPPH), Folin–Ciocalteu’s phenol reagent and Trolox were purchased from Sigma Chemical Co. (St. Louis, MO, USA). AAPH (2,2′-azobis (2-amidino-propane) dihydrochloride) and fluorescein sodium were purchased from Acros Organics (New Jersey, USA) and Panreac (Barcelona, Spain), respectively. The reagents used for chemical investigation (ethanol absolute anhydrous, chloroform, isooctane, acetic acid, Diethyl ether, n-Hexane, sodium hydroxide solution, sodium thiosulfate solution, acetic acid glacial) were purchased from Carlo Erba (Milan, Italy); potassium iodide was acquired from Honeywell Fluka (Steinheim, Germany), Amberlite^®^ XAD-7HP 20-60 mesh from Sigma-Aldrich (St. Louis, MO, USA) and Lecithin from Carlo Erba (Milan, Italy).

### 2.2. Sample Collection 

Olive Mill Wastewater (OMWW) is a secondary product of the olive oil extraction process containing soft tissues of the olive fruit and the water used in the various stages of the oil extraction treatment together with the water contained in the fruit. OMWW were obtained by mean of a three-phase centrifugation process from Ottobratica olive cultivar during the crop seasons 2019 and supplied by an olive oil mill located in the Calabrian region (Italy). Sunflower oil used for phenolic enrichment was purchased in a local market. 

### 2.3. OMWW Extraction 

In order to obtain an extract enriched of phenolic compounds, OMWW were processed with Amberlite XAD-7-HP resin following the literature [27] with some modifications. Amberlite XAD-7-HP resin have a large surface with a macroreticular structure that allows to recover a mixture of different sizes of polyphenols. Moreover its design and operation are simple, operating costs low and the resin regeneration is easy. Before performing the extraction procedure, the adsorbent was pre-conditioned with NaOH 0.1 N for 2 h, rinsed with distilled water, immersed in HCl 0.1 N for 2 h and finally washed with distilled water.

For the extraction, 50 g of OMWW sample were mixed with 10 g of resin under stirring for 20 min. The adsorbent was washed three times with water; successively, it was eluted by a mean of three volumes of 50 mL of EtOH. The combined ethanol extract (WE) was desolvented in a rotary vacuum at 25 ℃, then the dried residue was recovered with 100 mL of water, filtered using a 0.45-μm PTFE (Ø 15 mm) syringe filter and stored at 4 ℃ for the successive analytical determinations.

### 2.4. Production of Enriched Sunflower Oil

Enriched sunflower oil (MBoil) was produced in a Food Technologies laboratory of the Mediterranean University of Reggio Calabria (Italy) following the literature [28] with some modifications. An aliquot of WE and lecithin were added to sunflower oil and mixed for five hours until complete homogenization, in order to obtain oil samples enriched with a final concentration of 50 mg L^−1^ of hydroxytyrosol. Sunflower oil samples were used as control. Oil samples were kept in dark glass bottles (150 mL) at 10 and at 25 °C (three independent replicates for each thesis and time) and periodically analysed at different times (0, 15, 45 and 90 days of storage). 

#### Extraction of Antioxidant Compounds

Phenols of MBoil were obtained by liquid–liquid extraction using methanol and according to the method [29] opportunely modified. Five grams of oil were added with 2 mL of methanol:water (70:30) and 2 mL of hexane and mixed with a Vortex for 10 min. The hydro-alcoholic phase was separated from the oil phase in a refrigerated (NF 1200R) centrifuge apparatus (Nǜve, Ankara, Turkey) at 5000 rpm, 4 °C for 10 min. Hydro-alcoholic extracts (WE) were recovered with a syringe, filtered through a 0.45-μm nylon filter, diameter 15 mm (Thermo Fischer Scientific, Waltham, MA, USA) and utilised for the phenolic compounds quantification and antioxidant activity.

### 2.5. Determination of Total Phenol Content and Evaluation of Antioxidant Activity 

Total Phenol content (TPC) of WE was determined in accordance to referred method [12] with minor modifications. Briefly, an aliquot of diluted WE was placed in a volumetric flask and mixed with deionized water (20 mL) and Folin–Ciocalteau reagent (0.625 mL). Then, 2.5 mL of saturated solution of Na_2_CO_3_ (20%) were then added after 3 min and made up to the 25 mL with deionized water. Thereafter, the mixture was left to react for 12 h in the dark and at room temperature. Sample absorbance was measured at 725 nm using a double-beam ultraviolet-visible spectrophotometer (8453 UV–Vis, Agilent, Waldbronn, Germany).

For the determination of TPC of MBoil, 0.05 mL of WE were mixed with 0.300 mL of Folin reagent and 0.25 mL of deionised water and, after 4 min, with 2.4 mL of an aqueous solution of Na_2_CO_3_ (5%). The mixture was maintained in a 40 °C water bath for 20 min and TPC was determined at 750 nm. Quantification was performed by mean of a calibration curve obtained at gallic acid concentrations from 1 to 10 mg L^−1^. The results were expressed as mg of gallic acid equivalent 100 mL^−1^ of WE and mg gallic acid 100 g^−1^ of oil. 

The evaluation of antioxidant capacity of WE and MBoil was performed by DPPH and ABTS assays [30,31]. An aliquot of diluted WE (1:50) was mixed with DPPH solution (6 × 10^–5^ mM) to the final volume of 3 mL and left in the dark for 30 min. The absorbance decrement was measured against methanol at 515 nm using a spectrophotometer (8453 UV–Vis, Agilent, Waldbronn, Germany) at 20 °C. The radical scavenging activity was expressed as mmol Trolox 100 mL^−1^ of WE and µmol Trolox 100 g^−1^ sample of oil. Then, 7 mM ABTS and 2.4 mM potassium persulphate (K_2_S_2_O_8_) solutions were mixed for the ABTS assay and placed at room temperature for 12 h in the dark for stabilization. The resulting ABTS∙+ solution was diluted with ethanol to obtain a blue-green chromogen with an absorbance of 0.70 (±0.02) at 734 nm. Then, 10 μL of diluted sample were added to the radical solution up to 3 mL and after 6 min the absorbance was measured. The quenching of initial absorbance was plotted against Trolox concentration (from 1.5 to 24 μM) and the results were expressed as TEAC values (mmol Trolox 100 mL^−1^ of WE and µmol Trolox 100 g^−1^ of oil).

In addition, the antioxidant activity of MBoil samples was also analysed by ORAC assay according to our previous study [32]. The ORAC assay was carried out on VICTOR X2 2030 Multilabel Plate Readers (PerkinElmer, Boston, Massachusetts, USA) in 96-well black microplate (PerkinElmer, Boston, Massachusetts, USA) using a fluorescence filter with an excitation wavelength of 485 nm and emission wavelength of 520 nm. The mix for reaction consisted of 130 μL of fluorescein solution, 50 μL of AAPH solution and 20 μL of phenolic extract. The fluorescence was measured at 37 °C immediately after the addition of fluorescein (time 0) and measurements of fluorescence kinetic were taken every minute for 30 times until the relative fluorescence intensity was less than 5% of the initial value. Results were expressed as µmol Trolox 100g^−1^ of oil.

#### Identification and Quantification of Phenolic Compounds

Identification and determination of the principal bioactive phenolic compounds of WE and MBoil were performed by UHPLC in accordance with [32]. The UHPLC system consisted of an UHPLC PLATINblue (Knauer, Berlin, Germany) equipped with a binary pump system using a Knauer blue orchid column C18 (1.8 μm, 100 × 2 mm) coupled with a PDA--1 (Photo Diode Array Detector) PLATINblue (Knauer, Berlin, Germany). The Clarity 6.2 software was used. Before the injection, phenolic compounds of MBoil were extracted using a variation of method [33]. One millilitre of oil was extracted with 1 mL of a methanol:water (80:20, *v*:*v*) in 2 mL Eppendorf reaction tubes. The mixture was shaken vigorously for 1 min using a vortex and then centrifuged (Micro Centrifuge Model 1K15 SIGMA, Laborzentrifugen, Osterode am Harz, Germany) at 13,000 rpm for 10 min at 10 °C. The methanolic phase was filtered with 0.22-μm nylon syringe filters, diameter 13 mm (Thermo Fischer Scientific, Waltham, MA, USA). The flow rate was 0.4 mL min^−1^ and the injection volume 5 μL. Acidified water (pH 3.10) (A) and acetonitrile (B) were the mobile phases and the applied gradient was the following: 95% A and 5% B (0–3 min), 95–60% A and 5–40% B (3–15 min); 60–0% A and 40–100% B (15–15.5 min). Quantification was performed by external standards (1–100 mg L^−1^) and results expressed as mg kg^−1^ of sample.

### 2.6. Measurement of Chemical and Physical Properties of Oils

Free acidity (% oleic acid), peroxide value (mEq O_2_ kg^−1^) analyses and extinctions parameters K232 and K270 were performed according to Official and standard methods [34,35,36]. 

The moisture of samples was tested in an Electronic Moisture Analyser MA37 (Sartorius, Goettingen, Germany). The analysis was performed using 5 g of sample at 105 °C. The results were expressed as percentages.

#### Sunflower Oil Oxidative Stability in Accelerated Storage Test

OXITEST Oxidation Test Reactor (VELP Scientifica, Usmate Velate, MB, Italy) was used in order to evaluate the opposition to fat oxidation. This method is recognized by AOCS International Standard Procedure (Cd 12c–16) for the determination of oxidation stability of food, fats and oils [37]. The analysis consists of monitoring the oxygen uptake of the reactive constituent of food samples to determine the oxidative stability under conditions of accelerated oxidation. Briefly, 5 g of oil sample were distributed homogenously in a hermetically sealed titanium chamber; oxygen was purged into chamber up to a pressure of 6 bar. The reactor temperature was set at 90 ℃. These reaction working conditions allow obtaining the sample Induction Period (IP) within a short time. The OXITEST allows to measure the modification of absolute pressure inside the two chambers and, through the OXISoft™ Software (Version 10002948 Usmate Velate, MB, Italy), automatically generates the IP expressed as hours by the graphical method.

### 2.7. Statistical Analysis 

Results of the present study were expressed as mean ± *SD* of three measurements (*n* = 3). Multivariate and One-way analysis of variance with Tukey’s *post hoc* test at *p* < 0.05 were performed by SPSS Software (Version 15.0, SPSS Inc., Chicago, IL, USA). 

## 3. Results and Discussion

### 3.1. Water Extract Characterization 

**Figure 1:**
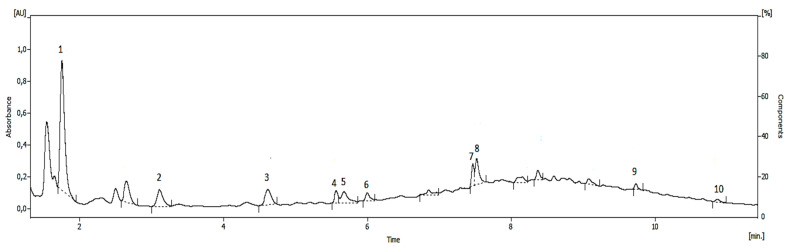
Chromatogram of phenolic compounds in wastewaters extract (WE). (1) hydroxytyrosol; (2) tyrosol; (3) chlorogenic acid; (4) vanillic acid; (5) caffeic acid; (6) *p*-coumaric acid; (7) verbascoside; (8) luteolin; (9) oleuropein; (10) apigenin.

As is well known, OMWW represent a complex medium in which more than 50% of the total phenolic components of the olive drupes are present [38]. UHPLC analysis provided identification of individual phenols in WE, as illustrated in Figure 1, but only the principal compounds were quantified (Table 1). The principal constituents of the Amberlite-desorption fraction were phenyl acids (caffeic, chlorogenic, *p*-coumaric and vanillic acids), phenyl alcohols (hydroxytyrosol and tyrosol), secoridoids (oleuropein), flavonoids (apigenin and luteolin), derivatives of hydroxycinnamic acid (verbascoside). WE showed a high content of hydroxytyrosol (834.51 mg 100 mL^−1^ of sample) and tyrosol (147.55 mg 100 mL^−1^ of sample) in agreement with other authors [39]. The TPC of WE was instead about 788.96 mg 100 mL^−1^ of sample. The amount of recovered phenolic compounds is related to interactions between the adsorbates and adsorbent as well as chemical structure of the compounds themselves. Non-polar resins, or weakly polar, such as XAD7HP, allow the recovery of low molecular weight phenols, especially when ethanol is used as desorbing solvent [15]. On the other hand, a high concentration of ethanol promotes the solubilisation of alcohol-soluble impurities resulting in a drop-in desorption capacity [40]. Considering the high initial phenolic concentration and the amount of soluble impurities in OMWW, an increased amount of adsorbed molecules could occur per unit mass of absorbent, leading to saturation and reduction of desorption yield. This could explain why the TPC value results lower than the sum of individual compounds quantified by UHPLC.

The antioxidant activity of WE was measured by mean of DPPH and ABTS^•+^ assays. Results obtained with the ABTS assay were higher as mmol TE 100 mL^−1^ than those obtained by DPPH, according to our previous study [32]. As reported in the literature, antioxidant activity is due to the synergism between the various phenolic compounds and the assay responses are affected by the functional group’s reactivity and characteristics, reaction time and complexity of the reaction kinetics [41,42].

### 3.2. Evaluation of Effect of WE on Sunflower Oil Stability

#### 3.2.1. Qualitative Parameters

In sunflower oil enrichment, lecithin was used in order to promote the dispersion of WE into the lipid matrix. Lecithin stabilizes the added phenolic compounds in the oil matrix due to its amphiphilic behaviour, producing reverse micelles that include the extract [28]. Firstly, the effectiveness of enrichment was evaluated in terms of qualitative parameters and oxidative stability. The characteristics of sunflower oil used in this work are shown in Table 2. The oil showed a low free acidity (0.05%) while 6.1 mEq O_2_ kg^−1^ of PV, according to the data about the commercial sunflower oil [43]. Spectrophotometric indices at 232 and 270 nm evidence the presence of dienes and trienes and the detected values (2.45 and 1.24) were characteristic of refined oils. Regarding enriched sunflower oil (Mboil), it is important to observe as the addition of extract involved the inclusion of water in the oil, as result of an increase in moisture content from 0.3% to 1% (Table 3). Free acidity of Mboil showed a rising trend during the storage: from 0.28 after enrichment to 0.32 at 10 °C and 0.35 at 25 °C after 90 days of storage, the formation of peroxides was reduced to about 49% in sunflower oil enriched with WE comparing with control sample at the beginning of storage. It could be linked to the phospholipids (the main constituents of soy lecithin) conferring to the oil oxidative stability [44]. According to the data in the literature [45,46,47], enriched samples, stored at different temperatures, showed a significant change (*p* < 0.01) of PV during storage. It can be noticed that the value of the PV was fluctuating progressively in time, reaching its maximum value on the 45th day (5.51 ± 0.21 at 10 °C and 5.47 ± 0.18 at 25 °C). The assessment of conjugated diene (K232) and conjugated triene (K270) is a reliable parameter for the measurement of oxidative deterioration of oils and, thus, the effectiveness of antioxidants in oils. The enriched samples had a little higher K270 values than the control; moreover, the conjugated triene content in the oil samples could be linked to the secondary oxidation compounds and conjugated trienes in the used commercial lecithin [48]. ANOVA data elaboration showed that no significant variations (*p* > 0.05) were observed during the storage at different temperatures. Likewise, other authors [46] have investigated the antioxidant efficacy of OMWW for the stabilization of lipid matrix obtaining the same our results. 

#### 3.2.2. Oxidative Stability

**Figure 2:**
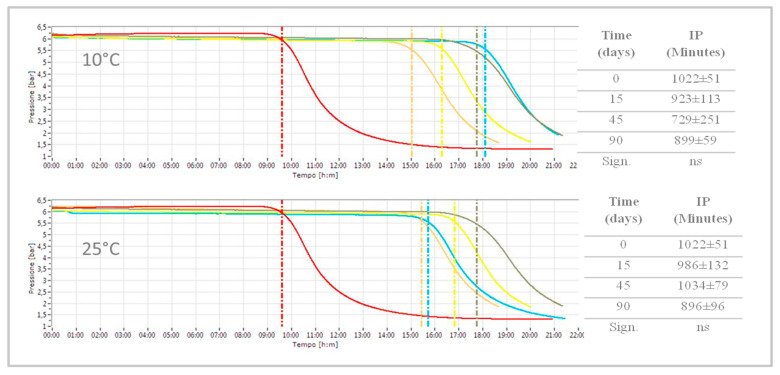
Oxidation curves during storage at 10 and 25 °C: red (sunflower oil), green (Mboil at 0 days), yellow (Mboil after 15 days), blue (Mboil after 45 days) and orange (Mboil after 90 days). ns: not significant.

To evaluate the resistance of fat oxidation, the oil samples were subjected to a high-oxidative stress environment using OXITEST reactor that shows a curve of oxidation characterized by an Induction Period (IP). It is the time necessary to reach an end point of oxidation that corresponds to a detectable rancidity or a rapid change in the oxidation rate. Oil stability was measured on control and Mboil just after the addition of WE and during storage, to evaluate the effect in protection from oxidation. In Figure 2, two examples of oxidation curves of oils stored at different temperatures were reported. The addition of the WE significantly involved an increase of the oxidative stability of the oils: Mboil samples had an average rise of oxidative stability of 50% (IP of 1022 min) with respect to the control that showed an Induction Period of about 540 min. The resistance of oxidation did not show a significant variation over time regardless of the storage temperature, from 0 to 90 days as reported in the tables included in Figure 2. The higher value of oxidative stability observed in enriched oils can be linked to the incorporation of phenolic compounds that are able to donate a hydrogen atom to the radical formed during the propagation phase of lipid oxidation [49]. Moreover, sunflower oil added with only lecithin was also analysed in order to evaluate the effect of the addition of lecithin on the sample stability against oxidation. From the Rancimat analysis of oil plus lecithin, a high Induction Period was observed compared with oil without emulsifier [50]. In our investigation, sunflower oil added with lecithin showed a lower value than control (420 min, Figure S1, Supplementary Materials). This confirms that the oxidative stability of enriched sample was related to the added phenolic extract. Previous studies also showed that the antioxidant protection of lecithin, attributed to phospholipids, was not effective for sunflower oil [51].

#### 3.2.3. Phenolic Composition and Antioxidant Activity 

The evolution of individual phenolic compounds added to oil was analysed during storage by UHPLC and the determination was repeated at different times (0, 45 and 90 days of storage). The analysis of the samples stored at different temperatures showed a similar phenolic composition (Table 4). According to the literature [52], samples were preferentially enriched with 50 mg L^−1^ of hydroxytyrosol. After the enrichment process, the chromatographic analysis of the samples showed (at time 0) a higher content of hydroxytyrosol and tyrosol, and a lower amount of caffeic acid, luteolin, oleuropein and verbascoside was also detected. In general, a significant decrease (*p* < 0.01) of phenolic compounds was observed during the storage, particularly after 45 days: about 59% of hydroxytyrosol and 32% of tyrosol were lost. Not significant variations were detected at the 90th day compared to the loss detected at 45th day, except for tyrosol, which showed another loss of 40% at the end of storage. In this regard, it is important to point out that authors ascribe to the amphiphilic character of lecithin the reduction of extraction yield of phenolic compounds due to the development of stable emulsion between lecithin and phenols [48]. This also could explain the low amount of phenolic compounds quantified compared to those added. In addition, we speculated that the formation of small drops of WE affected the reaction with Folin-Ciocalteau, underestimating results (Table 5).

The antioxidant activity of Mboil samples was analysed during storage using three different methods: ABTS, DPPH and ORAC assays. No single method is enough to determine the food antioxidant property, since different methods can give widely different results [53]. Moreover, the application of a single method can yield only a limited suggestion of the antioxidant activity of the samples under investigation [54]. The antioxidant activity of sample assays showed a significant variation (*p* < 0.01) over the time of storage of the enriched oils. A decrease of antioxidant activity was detected by ABTS assay according to the trend observed for TPC (*r* > 0.9). In contrast, a negative correlation was detected between TPC, DPPH and ORAC values. In addition, a non-linear trend was observed for DPPH and ORAC results with a minimum value detected at the 45th day. It should be considered that the added compounds have hydrophilic nature, thus their distribution in lipid phase is linked to their partition coefficient that may determinate also the distribution speed. It is conceivable that these compounds could be at first aggregated and only afterwards a suitable period distributed to the matrix. Multivariate analysis revealed that different temperatures did not significantly influence the total phenol content and the antioxidant activity measured by different assays while time seemed to affect them. Considering the oxidative stability of samples over time, the decrease of antioxidant activity is probably linked to the use of lecithin that led to the formation of lamellar structures in which hydrophilic antioxidants may be entrapped [48]. Despite this, from the start to the end of storage, at both temperatures, only about 3% of reduction was observed for the ORAC test: this is in line with the slow decrease of TPC in enriched oils and the observed oxidative stability of samples, so it confirms the robustness and the validity of ORAC method for the determination of the radical scavenging activity of the lipid matrix [50].

The obtained results confirm that the use of a low concentration of WE improve the nutritional quality of refined oil, whereas in previous studies the effectiveness of retarding lipid oxidation of oil is correlated to addition of high concentration of phenolic extract [9]. It is possible to hypothesize the use of WE for the fortification of different kinds of food products.

## 4. Conclusions

In conclusion, the addition of a phenolic extract obtained by OMWW in sunflower oil permitted the production of an enriched oil with a higher content of polyphenols and antioxidant properties for up to 90 days of storage. The two tested temperatures did not affect these results, so it can be considered an initial step to the enriched sunflower oil production, with increased antioxidant characteristics for a more prolonged time. The successful results are also linked to the valorisation of olive industry by-product, such as OMWW, converted from waste to resource, through an efficient methodology of extraction. 

## Figures and Tables

**Table 1:** Phenolic characterisation and antioxidant activity of wastewaters extract (WE). Data are expressed as mg 100 mL^−1^ for phenols and mmol TE 100 mL^−1^ for ABTS and DPPH assays.

Hydroxytyrosol	834.51 ± 0.71
Tyrosol	147.55 ± 0.70
Chlorogenic Acid	16.06 ± 0.70
Vanillic Acid	40.25 ± 0.17
Caffeic Acid	20.53 ± 0.47
P-Cumaric Acid	61.06 ± 0.71
Oleuropein	65.09 ± 0.67
Apigenin	74.62 ± 0.71
Verbascoside	876.91 ± 0.91
Luteolin	14.11 ± 0.89
Total Polyphenol Content	788.96 ± 1.41
ABTS	2569.19 ± 399.90
DPPH	114.37 ± 151.87

**Table 2:** Qualitative parameters of sunflower oil.

Free acidity (Oleic acid %)	0.05 ± 0.00
Peroxide value (mEq O_2_ kg−1)	6.10 ± 0.15
Moisture (%)	0.31 ± 0.01
Induction Period (minutes)	576 ± 0.01
K232	2.45 ± 0.09
K270	1.24 ± 0.08

**Table 3:** Qualitative parameters of Mboil during storage at 10 and 25 °C.

Temperature	Time (days)	Free Acidity (Oleic acid %)	Peroxide Value (mEq O_2_ kg^−1^)	Moisture (%)	K232	K270
	0	0.28 ± 0.02 ^b^	3.07 ± 0.03 ^c^	1.08 ± 0.14	2.49 ± 0.13	1.40 ± 0.01
	15	0.28 ± 0.03 ^b^	2.95 ± 0.13 ^c^	0.95 ± 0.03	2.57 ± 0.35	1.33 ± 0.20
**10 °C**	45	0.23 ± 0.03 ^c^	5.51 ± 0.21 ^a^	1.0 ± 0.9	2.55 ± 0.21	1.50 ± 0.04
	90	0.32 ±0.02 ^a^	3.85 ± 0.16 ^b^	1.0 ± 0.09	2.55 ± 0.21	1.50 ± 0.04
	Significance	**	**	ns	ns	ns
	0	0.28 ± 0.02 ^b^	3.07 ± 0.03 ^d^	1.08 ± 0.14	2.49 ± 0.13	1.40 ± 0.01
	15	0.28 ± 0.00 ^b^	4.32 ± 0.01 ^b^	1.04 ± 0.16	2.84 ± 0.17	1.42 ± 0.03
**25 °C**	45	0.23 ± 0.00 ^c^	5.47 ± 0.18 ^a^	1.80 ± 0.53	2.86 ± 0.39	1.40 ± 0.03
	90	0.35 ± 0.03 ^a^	3.78 ± 0.10 ^c^	0.77 ± 0.54	2.55 ± 0.21	1.37 ± 0.05
	Significance	**	**	ns	ns	ns

Different letters show significant differences among mean values by Tukey’s *post hoc* test. ** Significance at *p* < 0 01; ns: not significant.

**Table 4:** Phenolic compounds (mg kg^−1^ of sample) in Mboil during the storage (days) at 10 and 25 °C. letters and ** see Table 3.

**10 °C**	**0**	**45**	**90**	**Significance**
Hydroxytyrosol	39.65 ± 0.6 ^a^	16.25 ± 0.47 ^b^	15.06 ± 1.95 ^b^	**
Tyrosol	36.15 ± 0.16 ^a^	24.78 ± 0.5 ^b^	14.44 ± 1.23 ^c^	**
Vanillic acid	1.25 ± 0.01 ^a^	0.70 ± 0.02 ^c^	0.70 ± 0.01 ^b^	**
Caffeic acid	20.79 ± 0.59 ^a^	13.66 ± 0.02 ^b^	13.66 ± 0.00 ^b^	**
Verbascoside	9.11 ± 0.12 ^a^	8.74 ± 0.04 ^b^	8.92 ± 0.06 ^b^	**
Luteolin	18.19 ± 0.17 ^a^	12.19 ± 0.00 ^b^	12.24 ± 0.04 ^b^	**
Apigenin	11.05 ± 0.23 ^a^	4.59 ± 0.03 ^b^	4.69 ± 0.03 ^b^	**
**25 °C**	**0**	**45**	**90**	**Significance**
Hydroxytyrosol	39.65 ± 0.6 ^a^	15.77 ± 0.35 ^b^	15.88 ± 1.24 ^b^	**
Tyrosol	36.15 ± 0.16 ^a^	25.54 ± 0.32 ^b^	15.47 ± 1.29 ^c^	**
Vanillic acid	1.25 ± 0.01 ^a^	0.79 ± 0.04 ^c^	0.98 ± 0.04 ^b^	**
Caffeic acid	20.79 ± 0.59 ^a^	13.72 ± 0.02 ^b^	13.79 ± 0.00 ^b^	**
Verbascoside	9.11 ± 0.12 ^a^	8.73 ± 0.00 ^b^	8.87 ± 0.02 ^b^	**
Luteolin	18.19 ± 0.17 ^a^	12.19 ± 0.00 ^b^	12.18 ± 0.02 ^b^	**
Apigenin	11.06 ± 0.23 ^a^	4.61 ± 0.04 ^b^	4.74 ± 0.03 ^b^	**

**Table 5:** TPC (mgGA 100 g^−1^) and total antioxidant activity by ABTS, DPPH and ORAC assays (µmol TE 100 g^−1^) of the samples stored at different temperatures.

Temperature	Time (days)	TPC	ABTS	DPPH	ORAC
	0	37 ± 1 ^a^	1536.18 ± 1.55 ^a^	74.67 ± 2.79 ^b^	157.39 ± 0.86 ^a^
	15	26 ± 5 ^b^	1283.06 ± 4.93 ^b^	80.82 ± 2.50 ^a^	143.82 ± 0.88 ^c^
**10 °C**	45	23 ± 2 ^b^	1203.61 ± 7.69 ^c^	56.63 ± 1.66 ^d^	127.17 ± 0.91 ^d^
	90	23 ± 3 ^b^	1111.41 ± 9.78 ^d^	68.29 ± 0.88 ^c^	151.58 ± 1.22 ^b^
	Significance	**	**	**	**
	0	37 ± 1 ^a^	1536.18 ± 1.55 ^a^	74.67 ± 2.79 ^a^	157.39 ± 0.86 ^a^
	15	25 ± 3 ^b^	1285.05 ± 5.09 ^b^	68.29 ± 3.45 ^a^	153.21 ± 0.85 ^b^
**25 °C**	45	25 ± 2 ^b^	1240.37 ± 11.13 ^b^	55.19 ± 2.76 ^b^	128.90 ± 0.80 ^c^
	90	24 ± 2 ^b^	1204.21 ± 83.51 ^b^	69.58 ± 1.27 ^b^	152.15 ± 1.53 ^b^
	Significance	**	**	**	**

Means within a row with different letters are significantly different by Tukey’s post hoc test. ** Significance at *p* < 0.01.

## Supplementary Materials

The following are available online at https://www.mdpi.com/2304-8158/9/7/856/s1. Figure S1. Oxidation curves: red (sunflower oil), blue (sunflower oil plus lecithin), yellow, violet, orange and green (Mboil during the storage).
