# Supplementary material for: Impact of Stability of Enriched Oil with Phenolic Extract from Olive Mill Wastewaters
Source: Foods. 2020 Jun 30;9(7):856. doi: 10.3390/foods9070856 (PMC7404700; doi:10.3390/foods9070856)
Supplement: Supplementary file 1 [file foods-09-00856-s001.pdf]

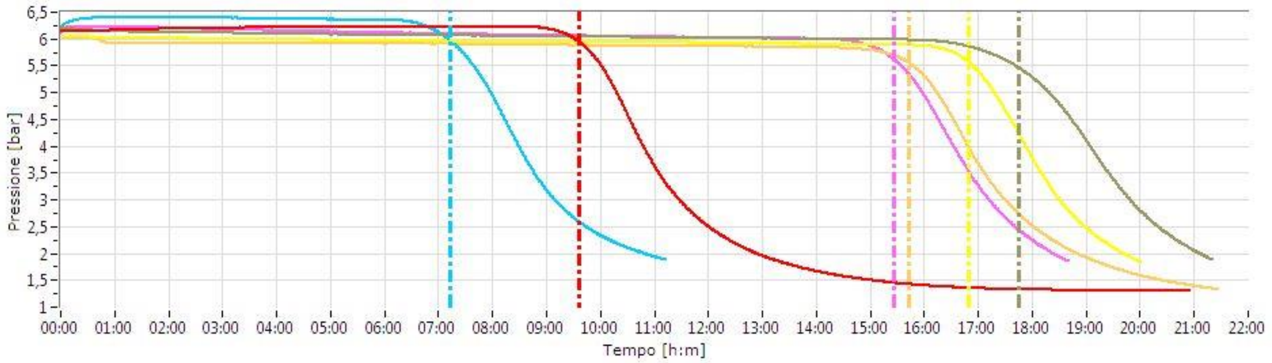

Samples	Time (days)	IP (minutes)	
sunflower oil	0	576	
oil plus lecithin	0	420	
mboil	0	1022	
mboil	15	986	
mboil	45	925	
mboil	90	915	

Figure S1. Oxidation curves: red (sunflower oil), blue (sunflower oil plus lecithin), yellow, violet, orange and green (Mboil during the storage).
